# Endometriosis of the Round Ligaments in Twins: A Rare and Unique Presentation

**DOI:** 10.5334/jbsr.3898

**Published:** 2025-04-16

**Authors:** Tom Oyen, Ragna Vanslembrouck

**Affiliations:** 1 UZ Leuven, Belgium

**Keywords:** endometriosis, inguinal canal, Nuck, twin, twins, mri, magnetic resonance imaging

## Abstract

*Teaching point:* The role of magnetic resonance imaging (MRI) in diagnosing endometriosis is growing, requiring radiologists to become familiar with both typical and atypical presentations of deep infiltrating endometriosis.

## Case History

**Figure 1:**
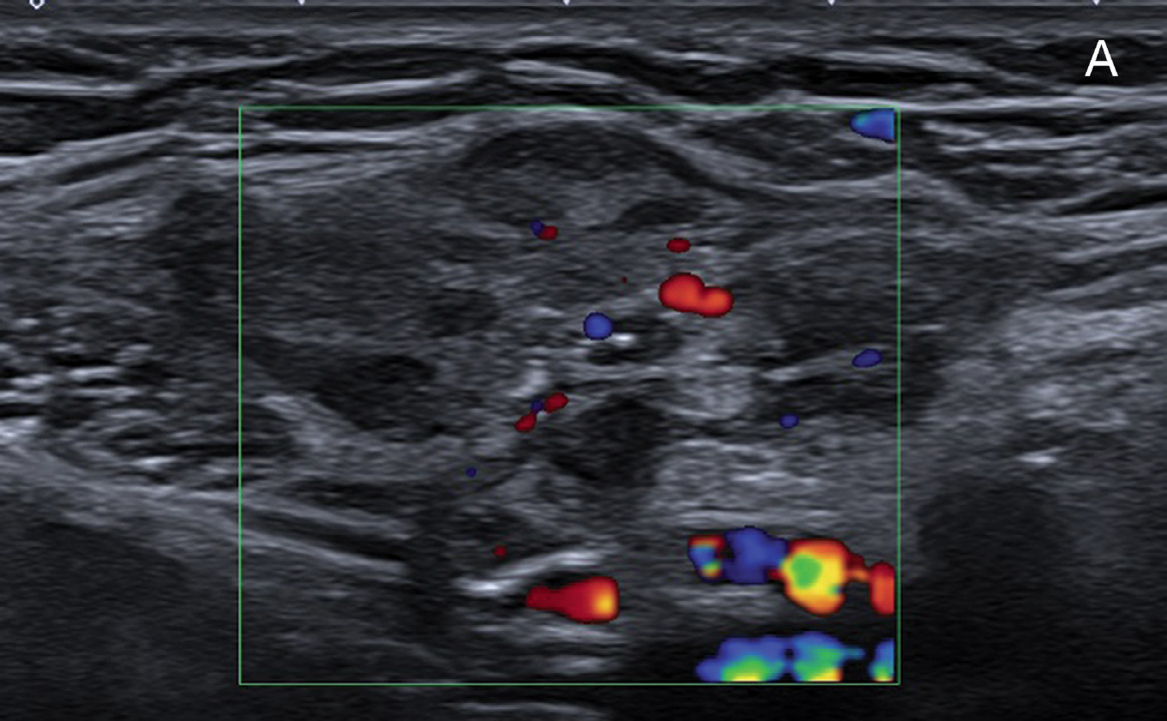

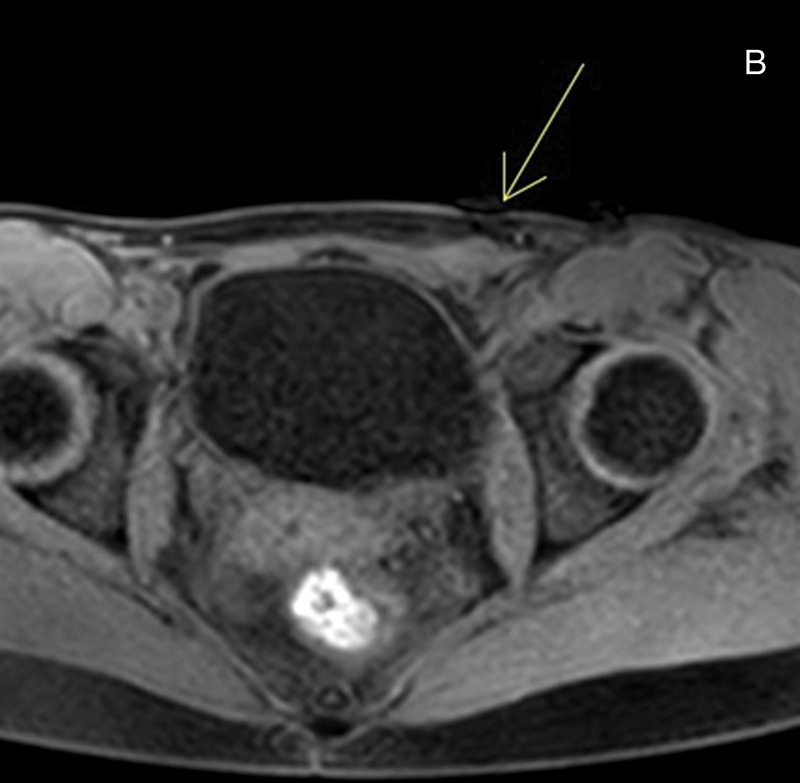

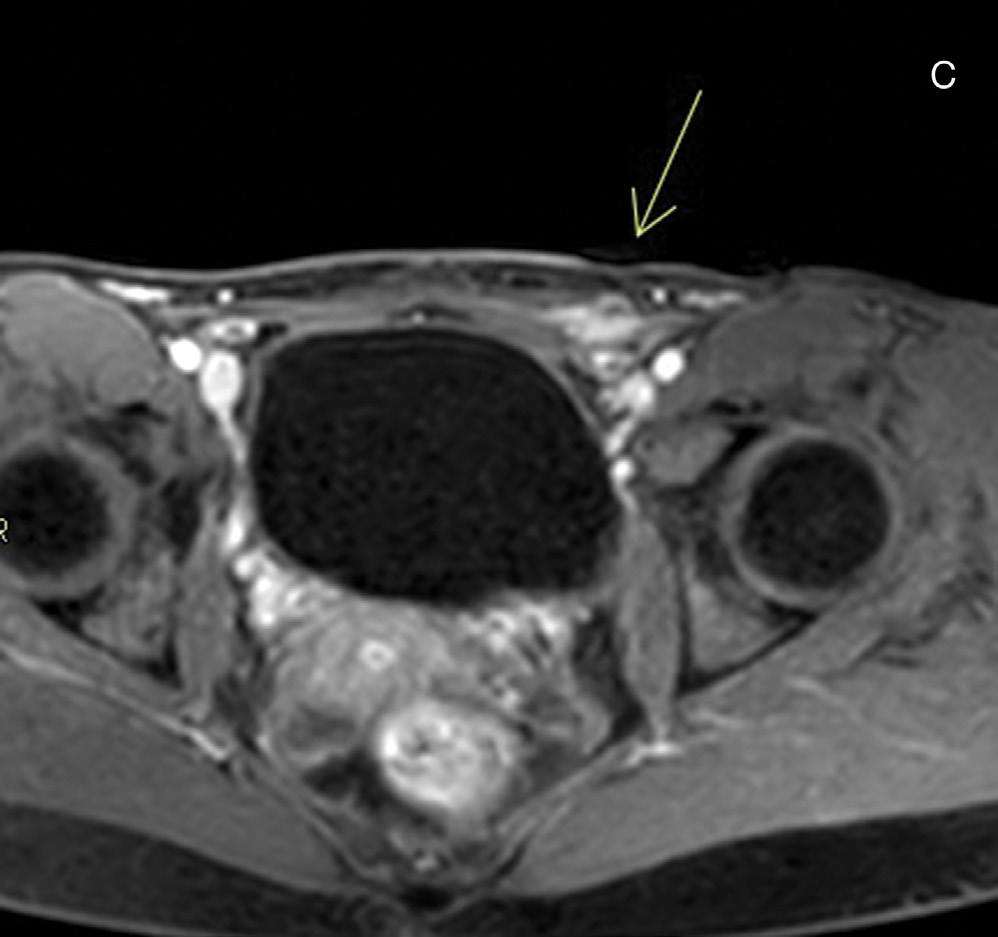
**A.** Ultrasound shows an mildly heterogeneous, hypoechoic mass with vascularity. **B.** Axial T1‑weighted fat suppressed image shows an isointense area in the left inguinal region. **C.** Same region as in Figure 1b, after intravenous administration of gadolinium, showing vivid lesional enhancement.

A 30‑year‑old woman presented with a right‑sided groin pain and swelling, not withheld on clinical examination. Ultrasound revealed a mildly heterogeneous, hypoechoic mass with vascularity. Magnetic resonance imaging (MRI) demonstrated a T1‑hypointense nodule in the left canal of Nuck with intense contrast enhancement (Figure 1). Biopsy confirmed the diagnosis of endometriosis.

**Figure 2:**
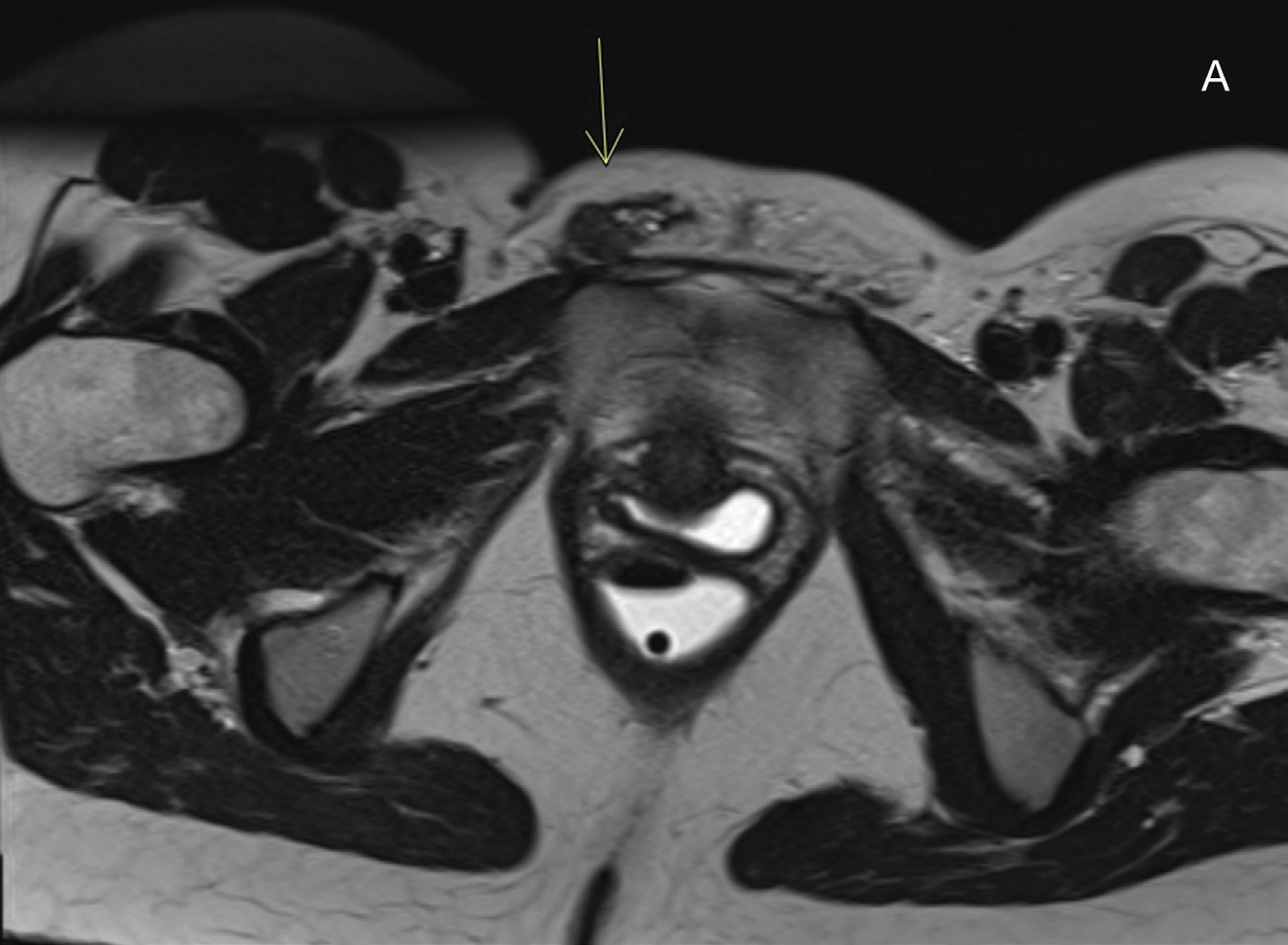

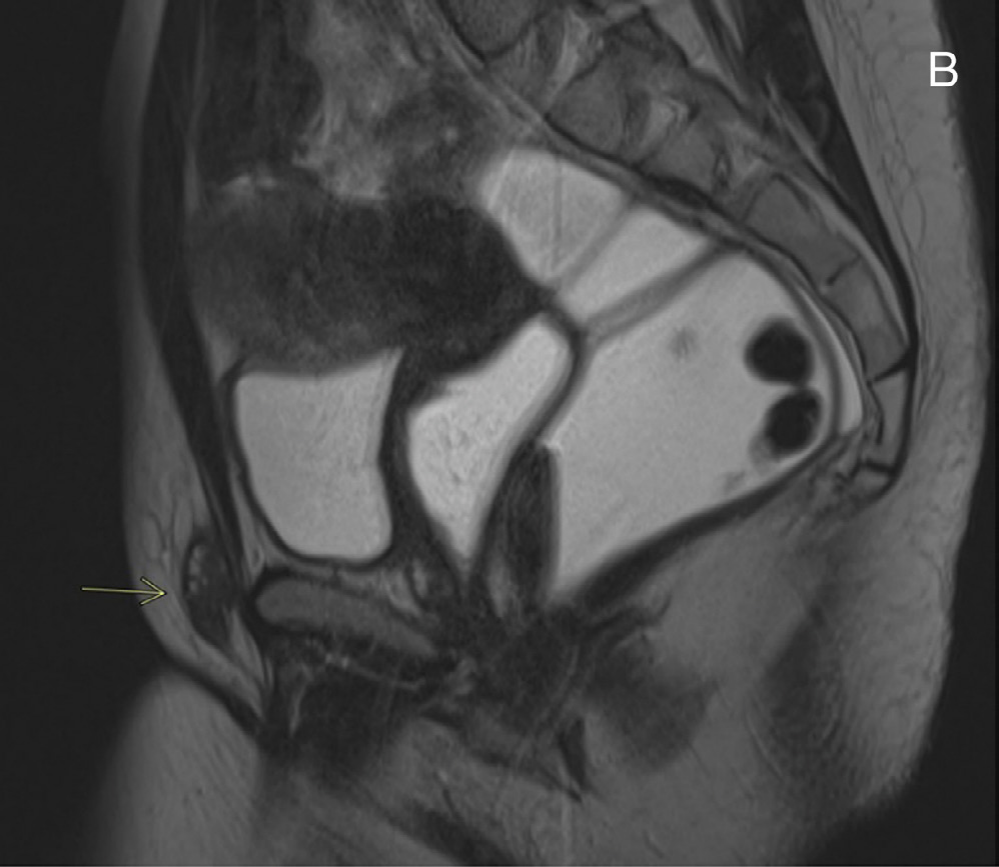

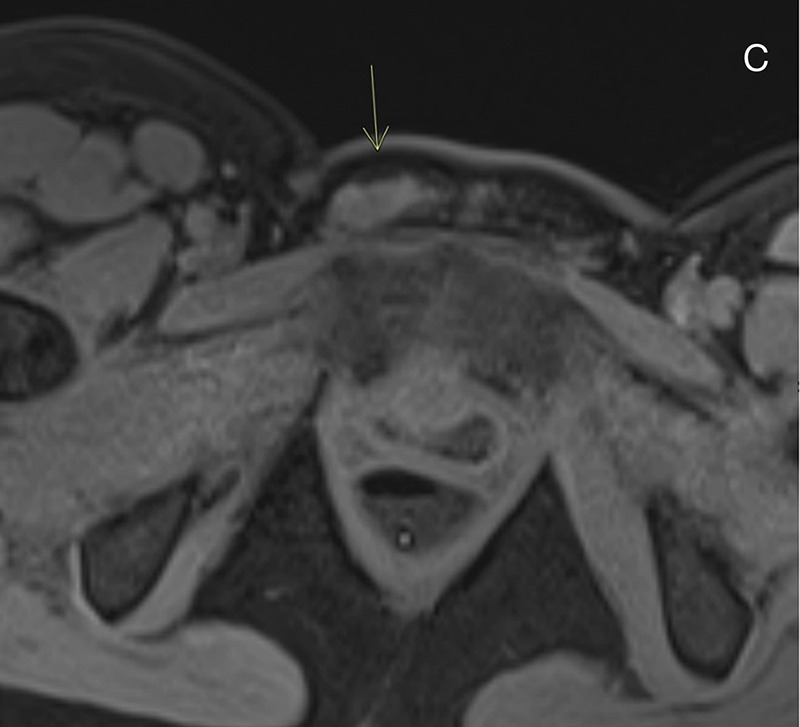
**A.** Axial T2‑weighted image shows a hypointense area in the right inguinal region with hyperintense foci. The mass is adjacent to the extrapelvic portion of the right round uterine ligament in the inguinal canal. **B.** Sagittal T2‑weighted image of the same area as Figure 2b. **C.** Axial T1‑weighted image with fat suppression shows the lesion with an isointense signal and discrete T1 hyperintense foci.

One year later, her identical twin sister presented with a swelling in the right groin that increased cyclically around the menses. A targeted MRI endometriosis protocol revealed a T2‑hypointense nodule with hyperintense glandular components extrapelvic around the right round uterine ligament, without T1‑hyperintensities, corresponding with endometriosis without hemorrhage (Figure 2).

## Comment

Endometriosis involving the round ligaments of the uterus (RLUs) is an uncommon manifestation, with a reported incidence ranging between 0.3% and 14%. This condition can affect either the intrapelvic or extrapelvic portions of the ligament, each presenting with distinct clinical and imaging features [1].

In extrapelvic involvement (canal of Nuck), patients typically present with a painful, palpable inguinal mass, which may or may not exhibit cyclical changes associated with menstruation. In intrapelvic involvement, symptoms are often nonspecific and can manifest as lower abdominal pain, posing a diagnostic challenge due to the lack of pathognomonic features [1].

MRI has become instrumental in identifying endometriosis of the RLUs, highlighting both morphological changes and signal alterations that differentiate normal from pathological tissue: normal RLUs are bilateral, thin, fibrous structures originating from the uterine horns, with low signal intensity on T1‑ and T2‑weighted sequences. Affected RLUs are thickened (>1 cm), shortened, and irregular, with nodular configuration on T2‑weighted images. Mixed lesions are most commonly observed comprising hypointense fibrous tissue interspersed with hyperintense hemorrhagic foci on T1‑weighted imaging [2].

Emerging research strongly suggests a significant genetic component in the development of endometriosis. Studies comparing concordance rates in monozygotic and dizygotic twins have consistently demonstrated a higher likelihood of disease occurrence in identical twins, indicating a substantial genetic influence [3].

One study estimated the heritability of endometriosis to be approximately 52%, further solidifying the role of genetic factors. Monozygotic twins often exhibit remarkable similarities in disease presentation, including shared lesion location and severity. Even dizygotic twins, while less genetically identical, tend to share common disease features, underscoring the combined impact of genetic and environmental factors [3].

Case reports of identical twins with endometriosis frequently highlight parallel disease trajectories, such as synchronized onset, symptom severity and affected anatomical sites. Intriguingly, one study reported nearly identical manifestations of deep infiltrating endometriosis (DIE) in dizygotic twins, further supporting the heritability hypothesis, even in individuals with less genetic similarity [3].
